# Polyamines Are Required for Virulence in *Salmonella enterica* Serovar Typhimurium

**DOI:** 10.1371/journal.pone.0036149

**Published:** 2012-04-30

**Authors:** Lotte Jelsbak, Line Elnif Thomsen, Inke Wallrodt, Peter Ruhdal Jensen, John Elmerdahl Olsen

**Affiliations:** 1 Department of Veterinary Disease Biology, Faculty of Life Sciences, University of Copenhagen, Frederiksberg, Denmark; 2 Center for Systems Microbiology, Department of Systems Biology, Technical University of Denmark, Lyngby, Denmark; Indian Institute of Science, India

## Abstract

Sensing and responding to environmental cues is a fundamental characteristic of bacterial physiology and virulence. Here we identify polyamines as novel environmental signals essential for virulence of *Salmonella enterica* serovar Typhimurium, a major intracellular pathogen and a model organism for studying typhoid fever. Central to its virulence are two major virulence loci *Salmonella* Pathogenicity Island 1 and 2 (SPI1 and SPI2). SPI1 promotes invasion of epithelial cells, whereas SPI2 enables *S*. Typhimurium to survive and proliferate within specialized compartments inside host cells. In this study, we show that an *S*. Typhimurium polyamine mutant is defective for invasion, intracellular survival, killing of the nematode *Caenorhabditis elegans* and systemic infection of the mouse model of typhoid fever. Virulence of the mutant could be restored by genetic complementation, and invasion and intracellular survival could, as well, be complemented by the addition of exogenous putrescine and spermidine to the bacterial cultures prior to infection. Interestingly, intracellular survival of the polyamine mutant was significantly enhanced above the wild type level by the addition of exogenous putrescine and spermidine to the bacterial cultures prior to infection, indicating that these polyamines function as an environmental signal that primes *S*. Typhimurium for intracellular survival. Accordingly, experiments addressed at elucidating the roles of these polyamines in infection revealed that expression of genes from both of the major virulence loci SPI1 and SPI2 responded to exogenous polyamines and was reduced in the polyamine mutant. Together our data demonstrate that putrescine and spermidine play a critical role in controlling virulence in *S*. Typhimurium most likely through stimulation of expression of essential virulence loci. Moreover, our data implicate these polyamines as key signals in *S.* Typhimurium virulence.

## Introduction

*Salmonella enterica* serovar Typhimurium (*S*. Typhimurium) is a Gram-negative facultative intracellular pathogen able to cause a wide variety of food- and water-borne diseases ranging from self-limiting gastroenteritis to systemic and life-threatening infections. Furthermore, *S.* Typhimurium causes a typhoid-like disease in susceptible mice, thus serving as an important model for studying the often fatal illness typhoid fever. The ability of *S.* Typhimurium to cause disease is largely dependent on two Type 3 Secretion Systems (T3SS1 and T3SS2) encoded by two distinct genetic loci named *Salmonella* Pathogenicity Islands 1 and 2, (SPI1 and SPI2), respectively [1]–[4]. Upon ingestion of *Salmonella* contaminated food, the SPI1 encoded T3SS1 injects a specific set of bacterial effector proteins into the epithelial cells lining the wall of the small intestines thereby promoting invasion of the host cells [5], [6]. Consequently, T3SS1 and its translocated effectors are essential for virulence in orally infected mice, but dispensable for systemic infections in intra peritoneal infected mice [1]. Following invasion of the epithelial cell layer *Salmonella* escapes to the underlying tissues [7] where it is taken up by phagocytes like macrophages and dendritic cells [8], [9] as reviewed in [10]. From here, it will rapidly spread through the lymphoid and blood systems to the spleen and liver resulting in a life-threatening systemic infection.

In both epithelial cells and macrophages, intracellular *S.* Typhimurium resides in a membrane bound compartment termed the *Salmonella* containing vacuole (SCV) inside which replication initiates. Within the SCV *Salmonella* uses T3SS2 to inject a specific set of effectors across the SCV membrane into the host cell cytosol that facilitates maturation of the SCV and SCV migration towards the Golgi [11]–[13]. Establishment of the SCV and intracellular survival are multi-factorial and depend on both SPI1 and SPI2 in addition to other factors such as fimbriae, flagella and ion transporters [11], [14]–[16]. Intracellular replication is primarily controlled by the SPI2 encoded T3SS2 and its secreted effectors [13], [17]–[19]. Accordingly, a functional T3SS2 system is required for the development of systemic disease in mice [4]. Expression of SPI1 and SPI2 is tightly controlled by multiple regulators organized in complex regulatory networks [20], [21], and are induced by both separate and common environmental signals [22]–[26].

Polyamines are small cationic amines present in all living cells. In bacteria, the predominant polyamines, putrescine and spermidine, are involved in a variety of functions including intercellular signaling, stress resistance and RNA and protein synthesis [27]–[29].

In contrast to *E. coli*, *S.* Typhimurium is unable to utilize putrescine and spermidine as sole sources of carbon and nitrogen [30] and the function of polyamines in *S.* Typhimurium remains largely unknown. Interestingly, it has recently been shown that polyamines play a central role in virulence of several intracellular pathogens including *Francisella tularensis*, *Legionella pneumophila*, and in *Shigella* spp. [31]–[35]. Additionally, in a comprehensive study of the intracellular gene expression profile of *S.* Typhimurium it was revealed that expression of the genes for putrescine and spermidine uptake is up-regulated during infection of epithelial cells and macrophages [36], [37]. This is suggestive of a role for these polyamines in both invasion and intracellular survival of *S.* Typhimurium. The present study was undertaken to investigate the roles of putrescine and spermidine in the virulence of *S*. Typhimurium. Our results reveal that these polyamines are essential for virulence of *S.* Typhimurium. Furthermore, our data demonstrate that these polyamines stimulate expression of both SPI1 and SPI2 genes, thus indicating that they function as key signals in the regulatory cascades controlling virulence gene expression in *S.* Typhimurium.

## Results

### Polyamines affects growth rate

**Figure 1:**
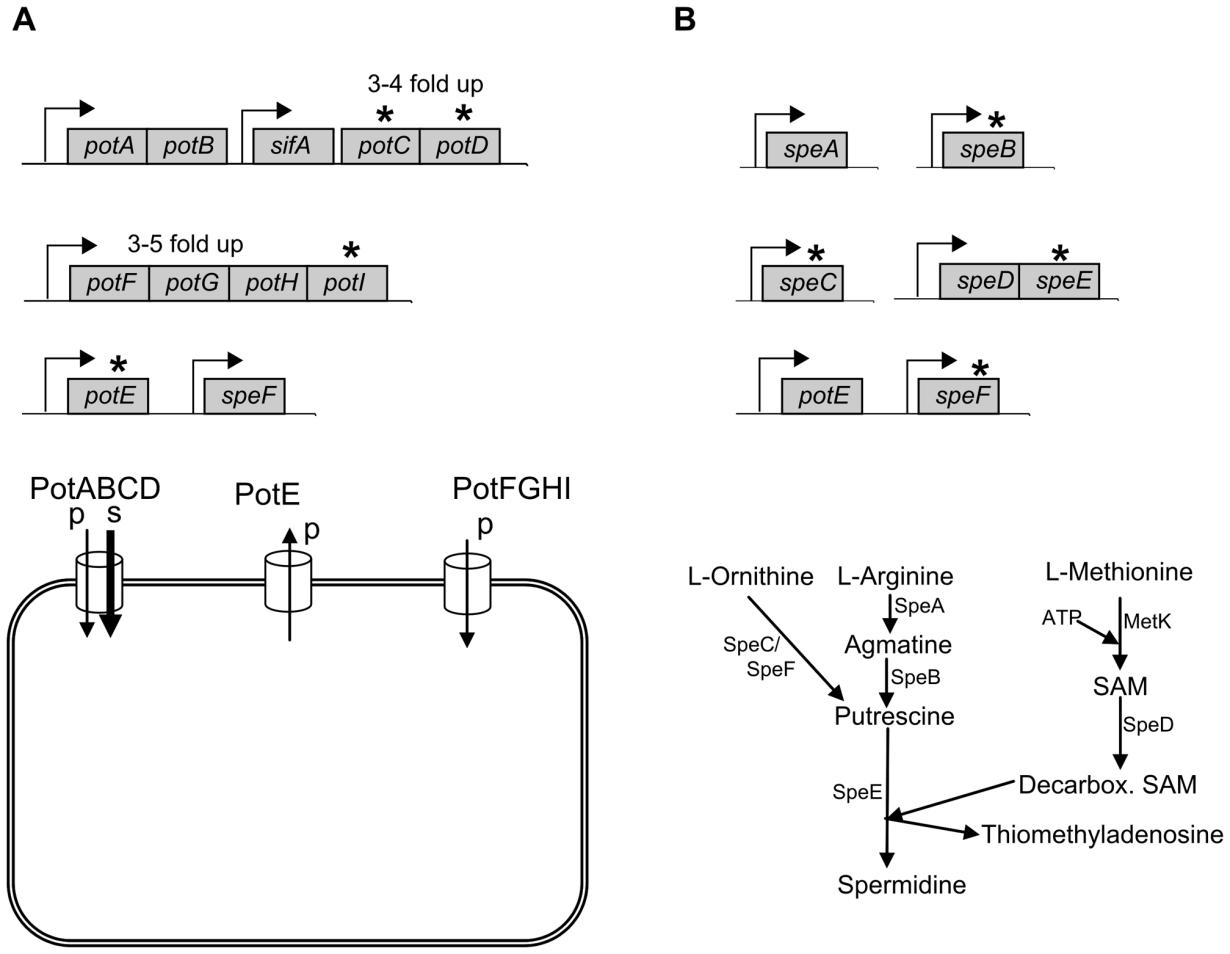
Gene organization and graphic representation of putrescine/spermidine transporters and biosynthesis pathways. (A) The putrescine and spermidine transporters are localized at three distinct loci; *potABCD* interrupted by *sifA*, *potFGHI*, and *potE*. The genotype of the transporter mutant (*potCD;I;E*) is indicated by an asterix above deleted genes. Below is shown a graphic presentation of the transporters with their substrate affinity indicated by p for putrescine and s for spermidine. Expression during infection of cell-cultures is indicated above genes [36], [37]. (B) The putrescine and spermidine biosynthesis genes are localized at five distinct genetic loci; *speA*, *speB*, *speC*, *speDE*, and *speF*. The genotype of the biosynthesis mutant (*speB;C;E;F*) is indicated by an asterix above deleted genes. Below is shown a graphic presentation of the biosynthesis pathways present in bacteria, reviewed in [67]. SAM: S-adenosylmethionine.

Polyamine content in bacteria is a function of active transport across the membrane and biosynthesis [38]. *S*. Typhimurium contains three conserved transport systems controlling putrescine and spermidine uptake (PotABCD and PotFGHI) and putrescine export (PotE) (Figure 1) [38]. Inside the bacterial cells, putrescine is synthesized from either L-Ornithine by the SpeC or the acid-inducible SpeF ornithine decarboxylases or from L-Arginine by SpeA and SpeB (Figure 1) [38]. Spermidine is synthesized from putrescine and L-Methionine by SpeE and SpeD [38]. To investigate the functions of putrescine and spermidine in *S*. Typhimurium we constructed two different mutants isogenic to the wt parent 4/74; a transporter mutant (*pot*-mutant) and a biosynthesis mutant (*spe*-mutant). In the genome of *S*. Typhimurium the *potABCD* operon has been interrupted by the insertion of the virulence gene *sifA* between *potB* and *potC* (Figure 1). Transcription of *sifA* is in the same direction as *potABCD*, indicating that deletion of *potCD* downstream of *sifA* rendering the transport system truncated should not affect *sifA* expression [39]. Hence, the *pot*-mutant carries deletions in *potCD*, *potE* and *potI* and is unable to export putrescine and import putrescine and spermidine. Importantly, expression of *sifA* was confirmed not to be affected in the *pot* mutant as measured by qPCR of RNA extracted from both the wt and the *pot* mutant (data not shown). In a recent study it was shown that deletion of the acid-inducible ornithine decarboxylase encoded by *speF* had no effect on virulence of S. Typhimurium [40]. However, the *spe*-mutant of this study, carries deletions in *speB*, *speC*, *speE* and *speF* and is impaired in biosynthesis of putrescine and spermidine (Figure 1).

**Figure 2:**
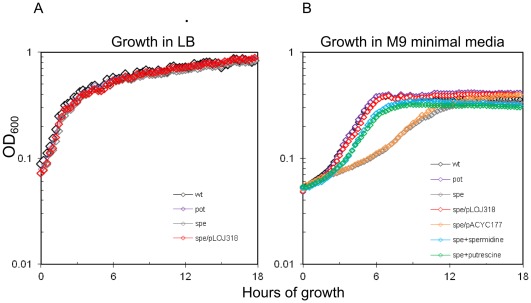
Growth *in vitro*. Indicated bacterial strains were grown at 37°C, 200 rpm for 16 hrs in either LB or M9 before sub-culturing into fresh media at a 40 fold dilution (LB or M9), and growth was followed every 15 min for 18 hrs using a Bioscreen C. Where indicated, the M9 was supplemented with either 100 µg ml^−1^ of putrescine or spermidine, respectively, during the growth experiment.

Initially the strains were tested for their ability to grow in rich media (LB) and in minimal media without polyamines (M9) (Figure 2A and B). In LB all strains grew similar (Figure 2A), whereas in M9, the *pot*-mutant (transport-mutant) grew similar to the wt and the *spe*-mutant (biosynthesis-mutant) had a slightly (approximately two-fold) reduced growth rate (Figure 2B). When the strains were incubated for growth overnight they all reached the same stationary level of growth. Addition of exogenous putrescine or spermidine to the media restored the growth rate of the *spe*-mutant. Similarly, growth was complemented by a plasmid carrying the *speB* gene restoring putrescine biosynthesis in the *spe*-mutant.

### Polyamines are required for invasion of epithelial cells

An initial step in *Salmonella* virulence is the adhesion to and invasion of the epithelial cells lining the walls of the small intestines. To investigate the role of putrescine and spermidine in adhesion, Int-407 human epithelial cells were infected with *S*. Typhimurium wt and the polyamine mutants. After 15 min of infection non-adherent bacteria were removed by washing and adherent bacteria were enumerated by plating on LB plates. Both mutants exhibited adhesion to epithelial cells similar to the wt indicating that lack of polyamines does not affect the adhesion of *Salmonella* to epithelial cells (data not shown).

**Figure 3:**
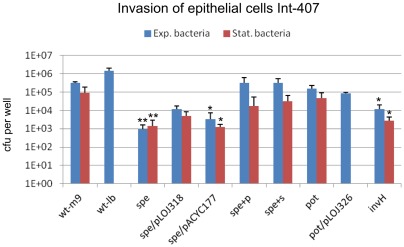
Invasion of epithelial cells. Int-407 cells were infected with exponential phase M9 cultures (blue bars) or overnight M9 cultures (red bars) of the indicated strains. Non-adherent bacteria were removed and adherent bacteria were enumerated by plating (not shown). For determination of invasion extracellular bacteria were killed by gentamicin and intracellular bacteria were enumerated by plating. The strains tested are: wt; *S*. Typhimurium 4/74, *spe* (biosynthesis), *pot* (transport), *invH*; SPI1 invasion mutant, spe/pACYC177; *spe*-mutant with blank complementation plasmid, spe/pLOJ318; *spe*-mutant complemented with *speB* (putrescine biosynthesis). pot/pLOJ326; pot-mutant complemented with potCD (spermidine/putrescine uptake). +p and +s denotes that the bacterial cultures have been supplemented with 100 µg ml^−1^ of putrescine or spermidine, respectively, prior to invasion. The experiments were repeated at least 4 times with similar results and shown is an average of these. Errorbars indicate standard deviations. Significant differences between the wt and the mutants are indicated with aterixs (* P<0.05; ** P<0.001). The P-values were calculated by a one-way ANOVA using Bonferronís post-test.

*Salmonella* invasion of non-phagocytic cells is mediated by SPI1 encoding T3SS1 and T3SS1-secreted effectors. The role of polyamines in invasion of epithelial cells *in vitro* was assessed in an Int-407 cultured cell invasion assay (Figure 3). Prior to infection bacteria were grown in M9 exponentially or in stationary phase as indicated. As a control, invasion of the wt grown exponentially in LB-media were also tested, as this has been reported to be optimal conditions for SPI1 induction [41]. Exogenous complementation of the *spe*-mutant was investigated by growing the mutant in M9 media supplemented with putrescine or spermidine prior to infection as indicated (Figure 3). After 15 min of invasion, extracellular bacteria were killed by gentamicin and intracellular bacteria were enumerated. The wt grown in LB had a 0.5-log higher invasion than the wt grown in M9, confirming that LB-media are better at inducing invasion than M9. Furthermore, exponential phase wt in M9 had 0.5 log higher invasion than when grown to stationary-phase. Independent of growth-phase prior to infection, the *spe*-mutant was significantly reduced, compared to the wt strain, in its ability to invade epithelial cells with invasion efficiency less than (exponential phase bacteria) or similar to (stationary phase) a SPI1 mutant (*invH*) (Figure 3). In contrast, the *pot*-mutant was not significantly affected in invasion. Invasion of the *spe*-mutant was improved (yet not significantly) by a plasmid carrying the *speB* gene restoring putrescine biosynthesis in the mutant. Interestingly, invasion of the *spe*-mutant could be fully complemented by growth in the presence of putrescine and spermidine, respectively, prior to infection. Together our results indicate that polyamines are required for expression or activity of an efficient invasion apparatus.

### Expression of SPI1 genes is reduced in the polyamine mutant

**Figure 4:**
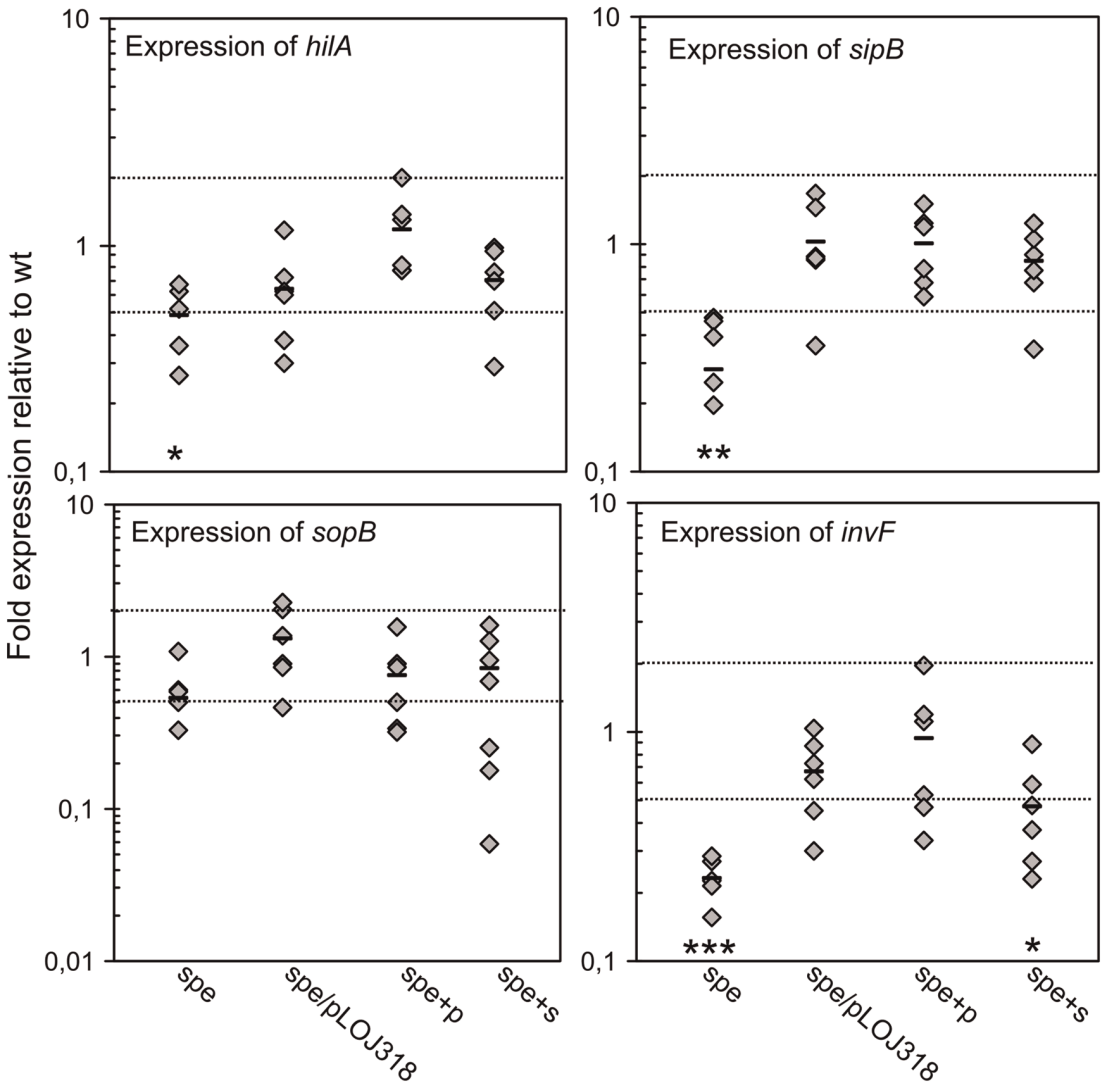
Expression of SPI1 genes. Relative expression levels of selected SPI1 genes in *S*. Typhimurium wt, spe-mutant and the complemented spe-mutant (spe/pLOJ318). RNA was extracted from overnight M9 cultures. +p and +s denotes that the bacterial cultures have been supplemented with 100µg ml^−1^ of putrescine or spermidine, respectively, prior to RNA extraction. A value of 1 indicates no detectable difference in expression between wt and mutant, values <1 indicate lower expression in the mutant and values >1 indicate higher expression in the mutant. Dotted lines mark the cut-off of two-fold regulation compared to wt. Results from 6 independent experiments are plotted for each strain. Outliers were removed using the Grubbs' test. Bar indicate average value of these for each strain. Significant differences between mutant and wt grown in M9 are indicated as follows: *** = P<0.0001; ** = P<0.001; * = P<0.05. The P-values were calculated by a one-way ANOVA using Dunnets post-test.

To investigate whether the impaired invasion of the *spe*-mutant was caused by reduced expression of the T3SS1 of SPI1 or reduced expression of T3SS1 secreted proteins we analyzed expression of *hilA*, *invF*, *sopB*, and *sipB* by qPCR (Figure 4) in the *spe* mutant grown in M9 and in M9 supplemented with either putrescine or spermidine as indicated. HilA is the master regulator of two separate SPI1 operons (*prg* and *inv/spa*) both encoding components of the T3SS1. *invF* is the first gene in the *inv*/*spa* operon and encodes an activator of *sipB* and *sopB* transcription [20]. SipB and SopB are effectors translocated by T3SS1. *sipB* is encoded within SPI1 and has a role in invasion and induction of host-cell death, whereas *sopB* is encoded distantly from SPI1 and has a role in invasion, SCV maturation and intracellular survival [14]. In the *spe*-mutant, expression of *sopB* was not significantly affected, however, expression of *hilA* was slightly, yet significantly, down regulated (Figure 4). Interestingly, expression of *invF* and *sipB* was both significantly 4-fold down-regulated, compared to the wt. The plasmid carrying the *speB* gene was able to partially complement expression of *hilA* and *invF* whereas full complementation was observed for *sipB*. Furthermore, growth in the presence of putrescine and to a lesser extent spermidine restored expression levels. Consequently, we conclude that putrescine and spermidine are required for full induction of some SPI1 genes thus providing a possible explanation for the polyamine dependent invasion observed earlier.

### Polyamines contribute to intracellular proliferation in epithelial cells

**Figure 5:**
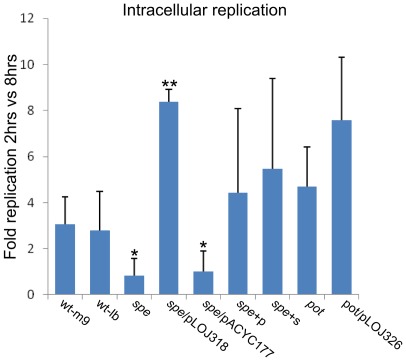
Intracellular survival/replication. Int-407 cells were infected with exponential M9 or LB cultures of the indicated strains of S. Typhimurium for 15 min at 37°. At this time point non-adherent bacteria were removed and extracellular bacteria were killed by gentamicin. To determine intracellular survival/replication intracellular bacteria were enumerated by plating after 2 hrs and 8 hrs, respectively. The strains tested are: wt; *S*. Typhimurium 4/74, *spe* (biosynthesis), *pot* (transport), spe/pACYC177; *spe*-mutant with blank complementation plasmid, spe/pLOJ318; *spe*-mutant complemented with *speB* (putrescine biosynthesis). pot/pLOJ326; pot-mutant complemented with potCD (spermidine/putrescine uptake). +p and +s denotes that the bacterial cultures have been supplemented with 100 µg ml^−1^ of putrescine or spermidine, respectively, prior to invasion. The experiments were repeated at least 4 times with similar results and shown is an average of these. Errorbars indicate standard deviation. Significant differences between wt grown in M9 vs other strains/growth conditions are indicated as follows: ** = P<0.001; * = P<0.05. The P-values were calculated by a one-way ANOVA using Dunnets post-test.

A central feature of *Salmonella* virulence is its ability to survive and replicate in host cells inside specialized compartments termed the *Salmonella* containing vacuole (SCV). The role of putrescine and spermidine in intracellular survival and replication was assessed by enumerating intracellular bacteria 2 hrs and 8 hrs post-invasion of Int-407 epithelial cells. In this assay, the wt had a 3-fold net-replication between 2 hrs and 8 hrs p.i. regardless of the pre-infection growth media (M9 versus LB) (Figure 5). The result is in line with previous observations of intracellular replication in epithelial cells where replication has been reported to initiate approximately 6 hrs post-infection [41]. The pot mutant was not affected in this assay, however, the *spe*– mutant was significantly reduced approximately 4-fold in intracellular survival compared to the wt with a net replication below 1 indicating that polyamines are important for intracellular survival and replication. Interestingly, net replication of the *spe*-mutant was enhanced above the wt-level by growth in the presence of putrescine and spermidine prior to infection signifying that pre-infection growth conditions affect intracellular survival of *S*. Typhimurium. Additionally, net replication of the *spe*-mutant was significantly enhanced above the wt level by a plasmid carrying the *speB* gene restoring putrescine biosynthesis in the mutant. These results indicate that the reduced intracellular growth of the *spe*-mutant is most likely caused by reduced induction of virulence in the absence of polyamines. Furthermore, the presence of exogenous polyamines prior to infection appears to enhance intracellular survival suggesting that polyamines function as an environmental signal that primes *Salmonella* for intracellular survival.

### Expression of SPI2 genes is reduced in the polyamine mutant

**Figure 6:**
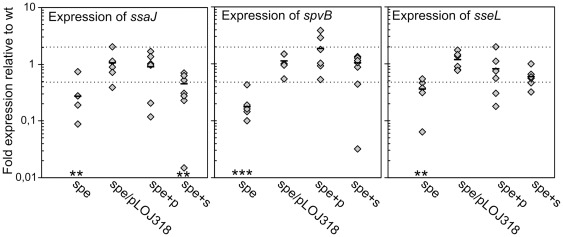
Expression of SPI2 genes. Relative expression levels of selected SPI2 genes in *S*. Typhimurium wt, *spe*-mutant and the complemented *spe*-mutant (spe/pLOJ318). RNA was extracted from overnight M9 cultures. +p and +s denotes that the bacterial cultures have been supplemented with 100 µg ml^−1^ of putrescine or spermidine, respectively. A value of 1 indicates no detectable difference in expression between wt and mutant, values <1 indicate lower expression in the mutant and values >1 indicate higher expression in the mutant. Dotted lines mark the cut-off of two-fold regulation compared to wt. Results from 6 independent experiments are plotted for each strain. Outliers were removed using the Grubbs' test. Bars indicate average value of these for each strain. Significant differences between mutants and wt grown in M9 are indicated as follows: *** = P<0.0001; ** = P<0.001. The P-values were calculated by a one-way ANOVA using Dunnets post-test.

To investigate whether the impaired intracellular survival of the *spe*-mutant was caused by reduced expression of T3SS2 or reduced expression of T3SS2 secreted proteins we compared the expression of *ssaJ*, *sseL*, and *spvB* by qPCR (Figure 6) in the *spe* mutant and the wt strain grown in M9 and M9 supplemented with either putrescine or spermidine. SsaJ is an essential component of the T3SS2 [42]. SseL and SpvB are effectors translocated by T3SS2. SseL is encoded within SPI2 and has a role in induction of host cell death [43]. SpvB is the second gene of the *spvABCD* operon encoded on the *Salmonella* virulence plasmid pSLT and is involved in SCV maturation and induction of host cell death [44]. Both *ssaJ* and *sseL* expression is activated by the two-component system SsrAB encoded within SPI2 whereas expression of *spvB* is controlled by SpvR also encoded on pSLT. In the mutant, expression of *ssaJ*, *sseL* and *spvB* were significantly 4-fold, 3-fold and 6-fold down regulated, respectively (Figure 6). The expression levels were partially restored by growth in the presence of spermidine and fully complemented by growth in the presence of putrescine and by the plasmid carrying the *speB* gene. In conclusion, polyamines are required for full induction of some of the T3SS2 genes and some of its effectors. Together with the reduced expression of SPI1 genes, the results provide a possible explanation for the observed intracellular phenotype of the *spe*-mutant (Figure 5).

### Polyamines are essential for virulence

**Figure 7:**
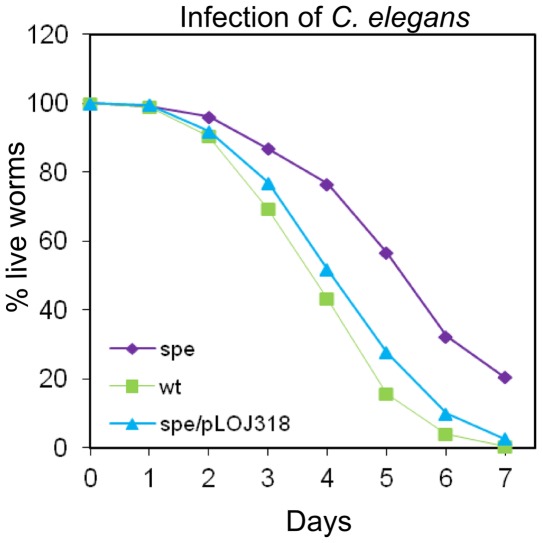
*C. elegans* killing assay. *C. elegans pha-1*(*e2123ts*) strain worms were seeded onto bacterial lawns on M9 plates of the wt (squares), the *spe*-mutant (diamonds) and the complemented *spe*-mutant (triangles) of *S.* Typhimurium. The plates were scored for live and dead worms every 24 hours. Three independent trials were performed for each strain and shown is an average of these experiments. Survival of the wt vs the spe-mutant was determined to be significantly different using the log-rank test for survival, p<0.0001.

Virulence of the wt and the *spe*-mutant was investigated in two animal models. Killing of the nematode *C. elegans* has previously been established as a model for studying *S*. Typhimurium virulence *in vivo* [45], [46] and is primarily dependent on SPI1 and the T3SS1 secreted effector *sipB* [47]. The *spe*-mutant killed *C. elegans* at a significantly slower rate than the wt (Figure 7). This correlates to the observed reduced expression of *sipB* (Figure 4). Additionally, the phenotype could be fully complemented by the putrescine biosynthesis plasmid (Figure 7). These results indicate that putrescine and spermidine are involved in SPI1 dependent virulence *in vivo*.

Competitive index (CI) tests [48] were used to assess the importance of putrescine and spermidine in the SPI2 dependent systemic infection of mice (Table 1), a well-described model for typhoid fever [49]. Both the *spe*-mutant versus the wt and the *pot*-mutant versus the wt had CIs that were significantly different from 1.0, indicating that polyamines are essential for systemic infection of mice. Accordingly, the CIs of the complemented *spe*-mutant and the complemented *pot*-mutant were significantly different than the CIs of the corresponding mutants (Table 1). In conclusion, these results show that polyamines are essential in *S*. Typhimurium for systemic infection of mice.

**Table 1:** Competitive index analysis of *S*. Typhimurium mutants.

wt versus	C.I.
*spe*	0.20±0.18^a^
*spe*/pLOJ318	0.63±0.15^b^
*spe*/pACYC177	0.36±0.11^a^
*pot*	0.17±0.15^a^
*pot*/pLOJ326	1.43±0.32^b^

The C.I. was calculated as the output ratio of mutant to wt divided by the input ratio. The C.I.s shown for the *spe* and the *spe*/pLOJ318 strains are the means of two independent infections of mice, with 4 mice in each infection group. The C.I.s of the other groups are the means of the 4-5 mice infected in each group. Mice were inoculated i.p. with a mixture of two strains comprising ∼5×10^3^ c.f.u. og each strain. Mouse spleens were harvested after 6 days for enumeration of bacterial c.f.u. The different strains used were differentiated on the basis of antibiotic sensitivity. Statistical differences were analyzed by one-way ANOVA using Bonferronís post-test.

a Significantly different from 1.0

b Significantly different from corresponding mutant.

## Discussion

### Polyamines are essential for virulence in *S.* Typhimurium

Polyamines are present in all living cells and in the intestinal lumen making them accessible upon ingestion and transfer of *Salmonella* to the small intestines and during intracellular proliferation. The recent reports that polyamines play a role in the virulence of several intracellular bacteria [31], [34], [35], and the finding that expression of polyamine uptake genes is up-regulated in *S*. Typhimurium during infection of mammalian cell cultures [36], [37], prompted us to hypothesize that polyamines might play an important role in host adaptation and virulence of *S*. Typhimurium. Accordingly, in this study, we show that a polyamine mutant of *S*. Typhimurium displayed defective invasion of epithelial cells, was reduced in intracellular survival/replication compared to the wt and was attenuated in the mouse model of typhoid fever. These results indicate that polyamines play an essential role for *Salmonella* during infection. The hypothesis was further supported by the fact that addition of putrescine and spermidine to the bacterial culture prior to infection of cell-cultures complemented the virulence phenotypes of the mutant. In conclusion, our results demonstrate that these polyamines are essential for both SPI1 and SPI2 mediated virulence of *S*. Typhimurium.

### Polyamines are required for induction of SPI1 and SPI2

The reduced virulence potential for the polyamine mutant was paralleled by reduced expression of both SPI1 and SPI2 genes. The expression of these loci is tightly controlled by multiple regulators operating at different levels in the respective transcriptional hierarchies leading to the correct spatio-temporal induction and level of SPI1 and SPI2 encoded proteins. Our results revealed, that the master regulator of SPI1, *hilA*, the t3ss1 *inv* operon and the *sip*-operon of SPI1 encoding SPI1 effectors and T3SS1 translocons were significantly down-regulated in the polyamine mutant. Furthermore, sampling of expression of SPI2 genes and the virulence plasmid, all contributing to intracellular survival, showed that expression of these genes were also significantly reduced (4–6 fold) in the polyamine mutant. Together these observations provide a possible explanation for the reduced invasion and intracellular survival of the polyamine mutant. Additionally, the reduced expression of virulence genes in the mutant is likely a contributing factor to the reduced virulence in the more complex model of typhoid fever.

### Are polyamines environmental signals for *Salmonella*?

Regulation of SPI1 and SPI2 expression is subjective to inputs from multiple environmental signals. Most of these signals are related to the environment *Salmonella* encounters during infection, i.e. oxygen tension, osmolarity, Mg^2+^ concentration, pH changes etc, reviewed in [20], [21]. However, induction of SPI2 in the intestines prior to invasion [50], [51] and the expression of SPI1 in response to diverse stimuli *in vitro* [41] indicate that additional un-identified signals affect expression of virulence genes in *Salmonella*. To our surprise, we found that the exogenous presence of putrescine and spermidine, in the culture media prior to infection significantly enhanced intracellular survival of the polyamine mutant indicating that *Salmonella* sense and respond to exogenous polyamines by transcriptional priming of the bacteria for intracellular survival. In support of this, it has recently been demonstrated that pre-invasion environmental factors influence *Salmonella*-host cell interactions [41]. Likewise, it has been shown that in *Pseudomonas aeruginosa*, *P. mirabilis* and *F. tularensis* exogenous polyamines function as environmental signals leading to altered bacterial gene expression [28], [33], [34], [52]. In *Salmonella*, the intricate regulatory networks controlling SPI1 and SPI2 expression are still not comprehensively described, but it is plausible that putrescine and spermidine could be affecting the expression of one or more of the controlling factors involved. In support of this hypothesis, expression of selected SPI1 and SPI2 genes was affected when the polyamine depleted mutant, was grown in M9 supplemented with putrescine or spermidine, however the putative genetic targets for the signal in *Salmonella* remains unknown. Both putrescine and spermidine are cationic amines capable of binding acidic molecules, like DNA and RNA, in the cells. In *E. coli* almost 90% of spermidine and 50% of putrescine is associated with RNA and accordingly it has been shown that polyamines through their binding to mRNAs alter their structure [53]. This structural influence on the mRNAs has in *E. coli* been shown to affect translation efficiency of specific targets including several important regulators [29], [54]–[56]. It remains a possibility that a similar polyamine dependent regulatory mechanism operates in *Salmonella*.

### Polyamines and bacterial virulence

Research reports from the last few years investigating the impact of polyamines in bacteria demonstrate that polyamines play diverse roles in modulating virulence in bacterial pathogens. For instance, in *Shigella* spp., which has diverged from its ancestral *E. coli* by uptake of a virulence plasmid, it was recently shown that it has a pronounced requirement for spermidine during infection [31]. This spermidine requirement has lead to parallel evolution-driven adaptive mutations silencing the *speG* gene encoding spermidine acetyltransferase, a spermidine metabolic enzyme, in all *Shigella* spp. In another example, intracellular growth of the water-borne pathogen *L. pneumophila*, is enhanced by host cells production of polyamines [35]. Interestingly, *L. pneumophila* has lost the genes for polyamine biosynthesis and therefore relies on polyamine up-take from the host-cell environment. In the present study, we have identified exogenous putrescine and spermidine as potential novel signals that modify *Salmonella* virulence end gene expression. Importantly, both SPI1 and SPI2 phenotypes and expressions are affected by polyamine depletion, indicating that polyamines function as a common signal required for the full induction of both loci. Our results add to the existing knowledge on the complex regulation of *Salmonella* virulence gene expression. Furthermore, together with the aforementioned reports that polyamines affect virulence in other pathogenic bacteria [28], [29], [33]–[35], [57], the results presented here point to that polyamines could function as small-molecule signals that modulate virulence and host adaptation of several bacterial species. Future studies on the roles of polyamines in *Salmonella* virulence are necessary to uncover novel regulatory mechanisms used by this intracellular pathogen to survive and spread inside its host.

## Materials and Methods

### Bacterial strains and growth conditions

Bacterial strains and plasmids are listed in Table 2. *S.* Typhimurium 4/74 was used as wild-type strain in all experiments. This strain has been described previously and its virulence is well defined [58]. The restriction deficient strain *S.* Typhimurium KP1274 was used as primary recipient for plasmids [59]. *S.* Typhimurium strains were maintained in LB media. For solid medium, 1.5% agar was added to give LB agar plates. Chloramphenicol (15 µg ml^−1^), kanamycin (50 µg ml^−1^) or carbenicillin (50 µg ml^−1^) was added as required. Prior to all experiments the bacteria were grown for 16 hrs, 200 rpm, 37°C in M9 minimal media (2 mM MgSO_4_, 0.1 mM CaCl_2_, 0.4 % glucose, 8.5 mM NaCl, 42 mM Na_2_HPO_4_, 22 mM KH_2_PO_4_, 18.6 mM NH_4_Cl) to deplete for carry-over polyamines. Where indicated, M9 was supplemented with 100 µg ml^−1^ of either putrescine (1.13 mM) or spermidine (0.7 mM). *Escherichia coli* Top10 competent cells were used for DNA cloning and were grown in LB media or on LB agar plates at 37°C.

**Table 2:** Strains and plasmids.

Strains and plasmids	Relevant characteristics	Reference
***S*** **. Typhimurium strains**		
4/74	Virulent reference strain	[58]
KP1274	Restriction deficient strain	[59]
LJ318	*ΔspeB; ΔspeC; ΔspeE; ΔspeF*, KnR, CmR	This work
LJ269	*ΔpotCD; ΔpotE; ΔpotI*, KnR, CmR	This work
LJ326	4/74/pACYC177, KnR	
LJ328	*ΔspeB; ΔspeC; ΔspeE; ΔspeF*/pLOJ318, Kn^R^, Cm^R^, Ap^R^	This work
LJ327	*ΔspeB; ΔspeC; ΔspeE; ΔspeF*/pACYC177 Kn^R^, Cm^R^, Ap^R^	This work
*invH* mutant	*invH*201::*TnphoA*	[68]
***E. coli*** ** strain**		
Top10	Cloning strain	Invitrogen
**Plasmids**		
pACYC177	Cloning vector, ap^R^, kn^R^	[61]
pKD46	Plasmid with λ-Red recombinase expressed from arabinose inducible promoter	[60]
pKD3	Template plasmid for λ-Red mutagenesis, Cm^R^	[60]
pKD4	Template plasmid for λ-Red mutagenesis, Kn^R^	[60]
pCP20	FLP plasmid for deletion of resistance cassette	[60]
pLOJ318	pACYC177 expressing the *speB* gene	This work

### Construction of strains and plasmids

Gene deletions and concomitant insertion of an antibiotic resistance cassette were constructed using Lambda Red mediated recombination as described elsewhere [60]. All constructs were verified by PCR and moved into a clean background via P22 phage transduction. Double/triple/quadruple mutant strains were also constructed by P22-mediated transductions. Primers used to construct mutants are listed in Table S1, supplementary material. In some cases, the antibiotic resistance cassette was removed by FLP-mediated recombination with introduction of pCP20 [60].

#### pLOJ318

A fragment containing the *speB* gene including 321 bp upstream region was PCR amplified with primers speBkompfwd and speBkomprev (Table S1), digested with *BamH*I and inserted into pACYC177 [61] opened with *BamH*I. The construct was verified by sequencing. The construct was used for genetic complementation of putrescine biosynthesis.

#### pLOJ326

A fragment containing the *potCD* genes was PCR amplified with primers potCfwd and potDrev (Table S1), digested with *BamH*I-*Hind*III and inserted into pACYC177 [61] opened with *BamH*I-*Hind*III. The construct was verified by sequencing. The construct was used for genetic complementation of spermidine/putrescine uptake.

### Infection of epithelial cells

Int-407 cells (HeLa-derived epithelial cells) were grown in MEM+ GlutaMAX^TM^-I, Earles, 25 mM HEPES (Gibco) supplemented with 10% (v/v) heat-inactivated fetal bovine serum (Invitrogen), in a humidified 37°C, 5% CO_2_ incubator. 24 hrs prior to infection, Int-407 cells were seeded in 24-well plates at 5×10^5^ cells per well. For stationary phase bacteria: *Salmonella* were grown for 16 hrs, 200 rpm, 37°C in M9 and collected by centrifugation at 6000 rpm for 5 min, resuspended to OD_600_ = 1.0 (1×10^9^ bacteria per ml) in 0.9% NaCl and added to monolayers at a multiplicity of infection of 100∶1. For exponential phase bacteria: *Salmonella* were grown for 16 hrs, 200 rpm, 37°C in M9 and sub-cultured in fresh M9 and grown for 3 hrs. Under these circumstances bacteria reached OD600∼0.5. At this point bacteria were collected by centrifugation at 6000 rpm for 5 min, resuspended to OD_600_ = 1.0 (1×10^9^ bacteria per ml) in 0.9% NaCl and added to monolayers at a multiplicity of infection of 100∶1. Monolayers were centrifuged at 1000 g for 5 min immediately after addition of the bacteria and then incubated for 15 min at 37°C, 5% CO_2_. Equal inoculum counts were checked by plating on LB agar plates. Extracellular bacteria were removed by aspiration and monolayers were washed twice with 0.9% NaCl. At this point (defined as time 0 hr) fresh media with 100 µg ml^−1^ gentamicin was added to kill extracellular bacteria. The plates were incubated for 1 hr at 37°C, 5% CO_2_ before media with 25 µg ml^−1^ gentamicin was added for the remainder of the experiment. To enumerate bacteria, cells were washed twice with 0.9% NaCl, lysed in 1 ml 1% Triton X-100 (v/v), 0.1% SDS (w/v) in PBS and bacteria were enumerated by plating on LB agar. For adhesion, bacteria were enumerated at t = 0 before addition of gentamicin. For invasion, intracellular bacteria were enumerated at t = 2 hrs. For replication/intracellular survival, intracellular bacteria were enumerated at t = 8 hrs.

### *C. elegans* killing assay

The virulence was assessed in *C. elegans* as previously described with modifications [62]. 20 μl overnight Salmonella culture in M9 containing 50 µg/ml kanamycin of each strain (LJ326, LJ318, LJ328) was spread onto M9 plates containing 50 µg/ml kanamycin and incubated at 37°C over night. For each strain, about 70 L4 hermaphrodites of the *pha-1* (*e2123ts*) mutant [63] were transferred from NGM plates seeded with *E. coli* OP50 to the plates seeded with *Salmonella* and incubated at 25°C. The plates were scored for live and dead worms every 24 hours. Three independent trials were performed for each strain.

### Mouse mixed infections

Female C57/BL6 mice (Nramp-) (20–25 g) were used to assess virulence of bacterial strains. Mice were inoculated i.p. with 0.1 ml of a 50∶50 mixture of wild type and mutated bacteria suspended in physiological saline. To prepare the inocula the wt, the *spe*-mutant and the complemented *spe*-mutant were grown for 16 hrs, 200 rpm at 37°C in M9 minimal media. Wild type and mutated strains were mixed before the infection to give a challenge dose of 5×10^3^ bacteria of each strain. The exact c.f.u. and ratio between wild type and mutated strains were enumerated by plating as described below. Mice were killed at 6 days post-inoculation by cervical dislocation. Severely affected animals were sacrificed early to this time point for animal welfare reasons, but otherwise treated as the rest of the group. The spleens were removed aseptically and bacteria recovered and enumerated after plating a dilution series on to LB agar. One hundred colonies were randomly picked and tested for resistance to the relevant antibiotic to determine the proportion of mutant strains. The competitive index was calculated as the mutant/wt ratio of the output versus the mutant/wt ratio of the inoculum. Mice experiments were conducted with permission from the Animal Experiments Inspectorate (http://www.dyreforsoegstilsynet.dk) in accordance with Danish law, license number: 2009/561–1675.

### RNA extraction and qPCR

Cells were grown in M9 or LB for 16 hrs, 200 rpm at 37°C. 1 ml aliquots were harvested and immediately frozen and stored at −80°C. Cells were lysed mechanically using the FastPrep system (Bio101; Q-biogene), and total RNA was isolated as described previously [64], [65] using the RNeasy mini kit (QIAGEN, Valencia, Calif.) according to the manufacturer's instructions. Total RNA was quantified by Nanodrop 1000 from Thermo Fischer. RNA was treated with Dnase (Fermentas) prior to use for qPCR. For qPCR the Maxima SYBR Green/ROX qPCR master mix (Fermentas) was used according to the manufacturer's recommendations. Primers used for qPCR are listed in Table S1 in supplementary material. As internal controls *nusG* and *rsmC* were employed giving similar results. These two genes had similar absolute expression levels in the wt and the *spe*-mutant (not shown). Fold regulation compared to the wt was calculated according to the method developed by Pfaffl [66].

### Statistical analysis

For multiple comparisons we used a one-way ANOVA with the indicated post-test. Outliers were removed using the Grubbs' test with a level of significance of 0.05.

## Supporting Information

Table S1 List of primers used in the study.
(DOC)
