# Supplementary material for: Polyamines Are Required for Virulence in Salmonella enterica Serovar Typhimurium
Source: PLoS One. 2012 Apr 30;7(4):e36149. doi: 10.1371/journal.pone.0036149 (PMC3340349; doi:10.1371/journal.pone.0036149)
Supplement: Table S1 — List of primers used in the study. (DOC) [file pone.0036149.s001.doc]

Table S1: Primers used in the study

| Primer name | Sequence 5´- 3´ |
| --- | --- |
| SpeBfwd | ACCTTAGGTCATCAGTACGATAACTCACTGGTTTCTAATGgtgtaggctggagctgcttc |
| SpeBrev | CTCGCCCTTCTTCGCCGCCTGGATATAGAGCATTTCTAATcatatgaatatcctccttag |
| SpeBcon | CAAACCCTTCCTCGTCGGGCCTAACGAC |
| speBconRev: | TTGCGCAAACGCGGTATCAGTCCGGCAG, |
| speBkompfwd | gaggatccGAGATGACCCTCATTCCCGGTAGCGGTC (BamHI) |
| speBkomprev | gaggatccATCAGCTGTAGGCCGGATAAGCGCAGCG (BamHI) |
| potCfwd | GCAAGCTTCCCCATCCTCTGAATAAGAAGGTGG |
| potDrev | GCGGATCCGCTTTGGCTGAATTAGCGTCCTGCT |
| SpeCfwd | TATTGCCGCCAGCGGTGAACTCATTCCCCGTCTCTCCACCgtgtaggctggagctgcttc |
| SpeCRev | TTAAAACATATCCGTACAAGCGTTGAATTCCATCGGCATCcatatgaatatcctccttag |
| SpeCcon | CTGATGTCTGGCGGTCGGAGCTGGTATC |
| speCconRev: | TCGGCTGTATCCTGGGCGGCATTGTGAG, |
| SpeEfwd | AATACGATGTGGCATGAAACGCTACACGACCAGTTTGGTCgtgtaggctggagctgcttc |
| SpeERev | GGACAGTGCGTCATGTAGATATTGCGGTAGCGCAAATGCAcatatgaatatcctccttag |
| SpeEcon | TGGCCCGCGATTCTCTGAGCCTGTTAGC |
| speEconRev: | CTGAAACCGAACGCCAGGAGATCACCGC, |
| SpeFfwd: | CATCGACGTGGCTGCCATCGTGTTATCCATTGACGACATTgtgtaggctggagctgcttc |
| SpeFrev: | CAGACCTGCTTACGGCCGTCGTGTTCTTCGATATAAACGCcatatgaatatcctccttag |
| SpeFcon: | CTGAATTTCCTGCAACCGGACGGGTTGGG, |
| SpeFconRev: | CCTCCGGTGAAGAAGCCATGCTGTACGG, |
| PotIfwd: | GGTTTACTTTTCTCTATGCGCCGATGTTAATGTTGGTGATgtgtaggctggagctgcttc |
| PotFGHIrev | GCCGCGTCTTGCACGCTGGATATCGCGAATCCGCTGTTcatatgaatatcctccttag |
| PotIcon: | CATGATTGGTCGCGTCCTGTGGCAGGAG, |
| PotCDfwd | CTCTAAAAGCACCCCATCCTCTGAATAAGAAGGTGGAACgtgtaggctggagctgcttc |
| PotCDrev | TGGTTTGCGCATCCGGATAGAGCGACTTATCATTGGCGcatatgaatatcctccttag |
| PotCDcon | CCTGCTGGCGCGCCGATATTATCGGTTC |
| PotEfwd | TCCAATAAAATGGGTGTCGTGCAGCTCACAATTCTGACgtgtaggctggagctgcttc |
| PotErev | AGCCGTGTTTATTTTTCAATTCAAAACGCGGTGAGACCcatatgaatatcctccttag |
| PotEcon | TATGCCTATCTGCGCGGCGAGGTCGAAC |
| Kanrev | CCGCTTCAGTGACAACGTCGAGCACAGC, |
| Camfwdny: | TACGCAAGGCGACAAGGTGCTGATGCCG, |
| qhilAfwd  qhilARev | AACACTGTACGGACAGGGCTATCGG  TACCATCGGGTATCATCTGCCCGGA |
| qsipBfwd  qsipBrev | AGGAGGCGACGGATCTCTATGAAGC  TCCGCTTTCTCGGCTTTCGCTTTGG |
| qsopBfwd  qsopBrev | ACTCAGCAGCAGGATGGCTTACCTG  TCATGCACACTCACCGTGGACATCC |
| qnusGfwd  qnusGrev | AAGAAGTCGTTGAGATCCGTGGCGG  GTTAAAGTCTGCGAACGGACCGTCG |
| qrsmCfwd  qrsmCrev | TGGCAGGTATTAAGCCGCCAGATGG  CGCGCGCTATCTACTTTGTTCAGCG |
| qinvFfwd  qinvFrev | ATGGCGCAGGATTAGTGGACACGAC  CAACGCTCTGCTGCACAAACGACGA |
| qspvBfwd  qspvBrev | CGCACGGAGAGCAGTTTTTATCGCC  GTACCTTGCTGAGATAGCGCATGGC |
| qssaJfwd  qssaJrev | GTAACCTTACGTGTCGAGCAGTCGC  GCCCTCCATCTGACTCAGCATTCCT |
| qsseLfwd  qsseLrev | TACGCAACGATGACCTGGGGCATAG  TAGAAAGGTCGGTTGTCGCGGAACC |
| qsifAfwd  qsifArev | GAGTTAGCCTGCGCATCGCAAAGAG  ATGTCGAAGGGAGGGGGTAGTCTAC |
